# An Evaluation of the Fear of Falling, Balance Levels, and Prognostic Blood Parameters Among the Geriatric Population With Hip Fractures

**DOI:** 10.7759/cureus.21704

**Published:** 2022-01-28

**Authors:** İsmail Gökhan Şahın, Emre Gültaç, Fatih İlker Can, Cem Yalın Kılınç, Nevres Hürriyet Aydoğan

**Affiliations:** 1 Orthopaedics and Traumatology, Muğla Sıtkı Koçman University Training and Research Hospital, Muğla, TUR; 2 Orthopaedics and Traumatology, Muğla Sıtkı Koçman University, Muğla, TUR; 3 Orthopaedics and Traumatology, Mugla Sıtkı Koçman University, Muğla, TUR

**Keywords:** geriatric injuries, prognosis, accidental falls, fear, hip fractures

## Abstract

Background

In this study, we determined that among patients who had been operated upon for hip fractures at our hospital, prognostic factors for mortality and functional recovery in the preoperative period were indicated via laboratory parameters using the International Falls Efficacy Scale (FES-I) and Berg Balance Scale (BBS) scores.

Methodology

Between January 2020 and January 2021, the results of 64 patients who had been surgically treated for a hip fracture and 57 patients who had scheduled elective surgery were compared retrospectively. The groups’ demographic data and blood parameters were compared. We used the FES-I and BBS scores to determine patients’ physical functional status and fear of falling.

Results

The case group’s statistically significant FES-I score was high, and its BBS score was low (p = 0.001/0.001). As expected, the case group’s D-dimer measurement was higher than the control group’s (p = 0.001). In addition, hemoglobin, platelet, lymphocyte, albumin, total protein, and calcium levels were lower in the case group (p = 0.001 for all levels). No significant difference was found for other parameters.

Conclusions

The scales are used by physical therapy, neurology, and orthopedics professionals to evaluate the geriatric population’s physical functional status and fear of falling. We believe prevention and cost-effective treatments for hip fractures can be achieved by determining geriatric patients’ hemoglobin, platelet, lymphocyte, albumin, total protein, and calcium levels upon hospital admission and by directing these patients to relevant clinics using the fear-of-falling and balance scales.

## Introduction

Hip fractures are a serious health problem that is common among the geriatric population [1]. The overall mortality rate within one year after a hip fracture has been reported to be 20-33% [1-3]. Meanwhile, 10% of patients cannot perform daily living activities alone after experiencing a hip fracture, with 19% needing long-term, continuous care [2,4]. The incidence of hip fractures increases with increasing life expectancy; this trauma’s treatment and care costs impose a serious burden on health systems [2-5]. Therefore, the prevention and cost-effective treatment of hip fractures are important.

In the literature, various studies have addressed the prevention of hip fractures, showing that increased age, comorbid diseases, and patients’ functional, physical, and mental status before a fall are associated with mortality [5]. Previous studies have also shown that falls are the dominant mechanism of injury for geriatric patients [6]. Therefore, preventative methods are important. Due to high fear-of-falling rates after a hip fracture among the geriatric population (21-85%), decreased physical activity levels and worsening functional results have been observed among these patients [1-3,7-10]. Studies have also reported that the fear of falling affects functional recovery more than pain [11].

Different opinions have been suggested in the literature regarding what causes the fear of falling, and the most frequently investigated factors are neuropsychiatric causes, namely, obesity, postural balance disorder, decreased muscle strength, and metabolic causes [1,3,7-10]. Post-traumatic symptoms, especially after falling, are common among geriatric patients. Neuropsychiatric symptoms, such as anxiety, depression, and delirium, are thought to be the most important factor in the fear of falling, causing post-traumatic symptoms after a fall [7].

In this study, we used International Fall Efficacy Scale (FES-I) and Berg Balance Scale (BBS) scores to determine the physical performance of geriatric patients who had been operated upon for hip fractures at our hospital, according to their pre-traumatic status [3,7,12,13]. To evaluate the fear of falling, laboratory parameters have been shown to be prognostic factors for mortality and functional recovery during the preoperative period [2,14,15]. This study aimed to compare geriatric patients who had planned for elective surgery with those who had scheduled surgery to treat a hip fracture, revealing differences between these groups and evaluating possible precautions for hip fractures in geriatric patients.

## Materials and methods

This study was approved by the Institutional Medical Ethics Committee of the Medical Faculty Hospital of Muğla Sıtkı Koçman University (2019/128). After receiving this approval, we included the results of 93 patients who had been surgically treated for hip fractures at our clinic and 60 patients who had planned elective surgery between January 2020 and January 2021.

Patients over 65 years of age were included in this study. The control group comprised patients who had scheduled arthroplasty due to coxarthrosis. Meanwhile, the case group comprised patients who had been admitted to the orthopedics department with a diagnosis of femoral pertrochanteric and neck fracture. Patients with a history of pre-surgical intensive care follow-up or a history of neurological diseases causing cognitive impairment and balance loss (Alzheimer’s, dementia, Parkinson’s, multiple sclerosis, stroke, vertigo, etc.) were excluded from the study.

Of the 93 patients in the case group, 14 had a history of neurological diseases (dementia (four), Alzheimer’s (three), Parkinson’s (three), stroke (three), and vertigo (one)), eight patients had a Mini-Mental State Examination (MMSE) score below 24, and seven postoperative patients had a history of follow-up at the intensive care unit. These patients were accordingly excluded from this study. Of the 60 patients in the control group, three patients had a history of neurological disease (Parkinson’s (two) and vertigo (one)) and were, therefore, excluded from the study. After these exclusions, the results of 64 patients who had been surgically treated for hip fractures and 57 patients who had planned elective surgery were compared retrospectively, and a total of 121 patients were included in this study.

We used the MMSE to determine patients’ cognitive levels [16]. Patients with a score lower than the cut-off value of 24 were excluded from the study. We also used FES-I and BBS scores to determine patients’ physical functional status and fear of falling. A score of 70 or higher for the FES-I and a score of under 45 points for the BBS were considered to indicate a high risk of falling [3,8,14-16]. The two study groups’ demographic data (age (years), height (cm), and weight (kg)) and preoperative hemoglobin (g/dL), platelet (10^3^/µL), lymphocyte (10^3^/µL), albumin (g/L), total protein (g/L), D-dimer (ng/mL), 25-hydroxycholecalciferol (vitamin D; ng/mL), serum calcium, and ionized and corrected calcium (mg/dL) levels, as well as their FES-I and BBS scores, were compared.

A post-hoc power analysis was performed after the study, and the working power was calculated to be 82%. Statistical analysis was performed using SPSS software version 21 (IBM Corp., Armonk, NY, USA), and the alpha error level was accepted as 5%. Because the normal distribution was not detected after the distribution analysis of the data, the median (minimum-maximum) values were used as descriptive statistics. Non-parametric data were compared using the Mann-Whitney U test, and categorical data were compared with the chi-square test.

## Results

Participants’ median age was 84 (61-94) years for the control group and 85 (61-95) years for the case group. Their median height was 165 (150-179) cm for the control group and 163.5 (153-178) cm for the case group. Meanwhile, participants’ median weight was 80 (68-105) kg in the control group and 80 (50-100) kg in the case group. No statistically significant difference was found between the groups in terms of demographic data (p = 0.730, p = 0.612, and p = 0.464 for age, height, and weight, respectively) (Table 1).

**Table 1:** Demographic data of the study participants.

	Control group			Case group			Mann-Whitney U test
	Median	Minimum	Maximum	Median	Minimum	Maximum	P-value
Age (year)	84	65	94	85	61	95	0.730
Height (cm)	165	150	179	163.5	153	178	0.612
Weight (kg)	80	68	105	80	50	110	0.464

FES-I and BBS scores were used to determine the physical functional status and fear of falling among participants [3,8,15-17]. While the median FES-I score was 38 (15-83) and the median BBS score was 26 (5-49) for the control group, the corresponding scores were 71 (31-100) and 6 (0-27) for the case group. A statistically significant difference was found between these groups’ scores (p = 0.001 for both the FES-I scores and BBS scores) (Table 2).

**Table 2:** Scale scores of the study groups.

	Control group				Case group			Mann-Whitney U test
	Median	Minimum	Maximum	Median		Minimum	Maximum	P-value
Fall Efficacy Scale (70)	38	15	83	71		31	100	0.001
Berg Balance Scale (45)	26	5	49	6		0	27	0.001

We also compared prognostic laboratory parameters [17]. The D-dimer measurement was statistically significantly higher for the case group (p = 0.001) compared to the control group, while hemoglobin, platelet, lymphocyte, albumin, total protein, and calcium levels were statistically significantly lower (p = 0.001 for all parameters). No significant difference was found between the groups’ vitamin D, corrected calcium, and ionized calcium levels (p = 0.054, p = 0.113, and p = 0.310, respectively) (Table 3).

**Table 3:** Prognostic blood parameters. Vit D: vitamin D; Hgb: hemoglobin; Plt: platelet; Lymp: lymphocyte; Ca: calcium

	Control group			Case group			Mann-Whitney U test
	Median	Minimum	Maximum	Median	Minimum	Maximum	P-value
D-dimer (0–500 ng/mL)	458	98	5,195	3,344	10	9,994	0.001
Vit D (20–50 ng/mL)	15.30	4.93	45.70	11.55	3.00	100.00	0.054
Hgb (11.2–19.9 g/dL)	12.9	7.7	14.9	9.8	6.1	18.4	0.001
Plt (180–370 10^3^/µL)	267	122	494	203.50	92	1,336	0.001
Lymp (1.18–3.74 10^3^/ µL)	1.770	0.50	4.51	1.125	0.37	20.00	0.001
Albumin (35–52 g/L)	42.50	26.6	47.8	32.30	20.0	47.0	0.001
Ca (8.6-10 mg/dL)	9.180	6.82	10.36	8.385	6.85	10.87	0.001
Total protein (64–83 g/L)	68.10	47.0	75.5	59.00	42.7	86.0	0.001
Adjusted Ca	9.042	7.75	10.10	8.970	8.10	11.10	0.113
Ionized Ca	2.7111	2.14	3.19	2.7725	2.06	3.81	0.310

## Discussion

Fear of falling is common among patients with hip fractures, worsening treatment outcomes [1-3,7-10]. The literature has offered different opinions on the cause of this fear of falling, and the most frequently investigated factors include neuropsychiatric causes, obesity, postural balance disorder, decreased muscle strength, and metabolic causes [1,3,7-10].

Although previous studies have shown that the fear of falling may be related to postural balance, no study has shown that the relationship between these two conditions is strong. Young et al. [11] reported that the fear of falling may be mainly associated with psychiatric causes. São Romão Preto et al. [18] found no relationship between the fear of falling and body composition, but they observed a more severe fear of falling among patients with low muscle strength in a “hand-grip” dynamometry test’s muscle strength measurements, which was associated with decreased function in muscle strength and balance between the arm and leg muscle groups. Meanwhile, Mak and Pang [19] concluded that both primary and recurrent falls are statistically significantly more common among patients with a fear of falling. Finally, Clague et al. [20] stated that anxiety causes fear of falling by creating hyperventilation and hypocapnia and, therefore, that fear of falling occurs due to neuropsychiatric causes.

In this study, we used FES-I and BBS scores to determine participants’ fear of falling and physical functional status. The median FES-I value for the case group was higher than 70, and it was significantly higher than the control group’s corresponding value. Moreover, we found that the case group’s BBS score was significantly lower than the control group’s; however, both groups’ median BBS scores were lower than the cut-off value of 45. Scores with a BBS score of less than 45 were considered to be at a high fall risk [12,13,21]. BBS scores were stratified, and changes in scores indicated clinical improvement during follow-up when investigated in each stratum, as suggested by Donoghue et al [13]. According to Donoghue et al. [13], patients with a score above 45 were considered normal, while patients with a score of 35-44 had at least five points indicating clinical improvement, patients with a score of 25-34 had at least seven such points, and patients with a score of 0-24 needed an increase of at least five such points. As a result, BBS was stratified into four groups [12,13,21]. In this study, both groups’ median values were lower than 45, but the difference between groups’ median values was 20. In our view, this difference in our study is meaningful according to Donoghue et al. [13].

Sim et al. [22] stated that a preoperative hemoglobin level of less than 10 g/dL among patients with a fractured hip was associated with a poor functional outcome. Meanwhile, Sheehan et al. [23] conducted a systematic review of anemia, showing that anemia’s effect on a hip fracture prognosis was weak. Hagino et al. [15] divided a total of 205 patients into two groups according to their movement (ambulatory) levels after receiving hip fracture treatment and compared the ambulatory and non-ambulatory groups in their published results. According to this study, age, dementia, anemia, a fluid-electrolyte imbalance, abnormal lung function, chronic systemic diseases, and home-care duration were found to be significantly higher among the non-ambulatory group. Hagino et al. found no significant difference between the groups regarding liver function, kidney function, or inflammation markers.

In this study, lymphocyte, platelet, albumin, total protein, and serum calcium levels, which are indicators of nutrition and liver function, were significantly low among the case group while D-dimer levels were high among the case group, in contrast to the results of Hagino et al. [15]. However, similar to the results of Hagino et al., anemia levels were significantly higher (low hemoglobin levels) among the case group [15]. We found no significant difference in corrected calcium or ionized calcium values. Vitamin D deficiency and insufficient calcium intake are common among the geriatric population, predisposing the elderly to hip fractures after a fall due to osteoporosis [24,25]. In this study, we expected high D-dimer levels because of the acute phase reactant for the case group, and we expected low vitamin D and corrected and ionized calcium levels for the case group; however, no statistically significant difference was found between the groups (p = 0.001 and p = 0.054, respectively).

## Conclusions

The FES-I and BBS scales are used by physical therapy, neurology, and orthopedics professionals to evaluate physical functional status and fear of falling among the geriatric population. We believe that hip fractures can be prevented and cost-effectively treated by determining geriatric patients’ hemoglobin, platelet, lymphocyte, albumin, total protein, and calcium levels upon their hospital admission and directing them to relevant clinics using the fear of falling scales.
